# Institutional Housekeeping

**Published:** 1911-12-09

**Authors:** 


### INSTITUTIONAL HOUSEKEEPING.

**(Contributions invited: Workers' Questions will be welcome.)**

"Temporary Matron" is thanked for her kind appreciation of our housekeeping articles. With regard to the amount of butter, etc., which ought to be used per head at the nurses' table, all depends on the nature of the meals, and it is unwise to lay down any hard-and-fast rule. It has long been found more economical to let everyone have as much as they want, rather than fix a stated quantity which each person is expected to consume. In estimating an allowance, the amount has to be regulated according to the appetites of those who take most, and thus a generally wasteful consumption of common necessaries is encouraged. To reduce the consumption of butter more variety should be introduced into the bill of fare. Too much bread and butter is monotonous, and when the bread used is white it is a very constipating diet also. To vary it, Quaker or Plasmon oats, very thoroughly cooked, should be served for breakfast with hot milk, and golden syrup or preserves should find a place on the table. A dish of freshly cooked rock buns, which can be prepared from clarified dripping, would make a pleasant feature at tea, and for supper the cold meat and bread and butter régime should be replaced by hot savoury dishes of macaroni, haricot beans, lentils, etc., served with boiled rice or baked potatoes. Most of these dishes can be prepared in the morning and just put in the oven to get hot, so that there is no additional strain on the cook in having a hot supper. To get attractive meals in a hospital without a nursing home the effort should be to have every article which appears on the table good of its kind, however simple the food. It is, of course, necessary to simplify both menus and service as far as possible, but this will not be resented if the food is hot and appetising. There need be no distinction between the sisters' and nurses' tables.

